# Mastering Midface Rejuvenation: A Proposal for a New Three‐Layer, Ultrasound‐Guided Technique

**DOI:** 10.1111/jocd.70664

**Published:** 2026-01-14

**Authors:** Paola Molinari, Simone Ugo Urso, Chiara Faso, Ilenia Iacovone, Giovanni Mosti

**Affiliations:** ^1^ Private Practice Modena Italy; ^2^ Sassari University Sassari Italy; ^3^ Private Practice Zola Predosa Italy; ^4^ Department of Anesthesia and Critical Care University of Florence Florence Italy; ^5^ Private Practice Lucca Italy; ^6^ Clinica MD Barbantini Lucca Italy

**Keywords:** facial ultrasound, fillers, skin booster, skin layers, three‐layer, three‐layer injection

## Abstract

**Introduction:**

Aging affects all facial layers: deep fat atrophy reduces midface projection, superficial fat changes alter facial contour, and dermal thinning compromises skin texture. Midface rejuvenation aims to restore malar projection, cheek volume, and youthful contours, thereby improving the nasolabial fold and suborbicular region. Conventional multilayer techniques address both deep and superficial fat compartments using fillers with distinct rheological properties, but often neglect skin quality and the advantages of ultrasound guidance. The approach we propose is grounded on three core principles: the comprehensive anatomical knowledge, the use of high‐frequency ultrasound to ensure precise and safe injections, and the selection of hyaluronic acid fillers specifically tailored to each tissue plane—deep, superficial, and dermal. The aim of our work was to present our technique as a proof of concept for a three‐layer, ultrasound‐assisted technique, which enhances safety, precision, and aesthetic effectiveness, representing a significant advancement in modern facial rejuvenation.

**Methods:**

A three‐layer, ultrasound‐guided midface rejuvenation technique employs tailored hyaluronic acid fillers for each plane. Firm, high G′ fillers target deep compartments through supraperiosteal injections for structural support. Elastic, moldable fillers, directly injected into superficial fat, enhance superficial layers while preserving natural mobility. Non‐cross‐linked formulations, used subdermally, by using a cannula, hydrate and improve skin quality, ensuring precise integration with minimal risk.

**Conclusions:**

The multilayer technique combines different fillers in deep, superficial, and dermal layers, tailored to the patient's anatomy. This three‐layer method restores volume and preserves natural expression, simultaneously improving skin quality, particularly benefiting hollow or mature faces. Unlike single‐plane injections, it addresses all aging aspects. Although not yet routine, ultrasound integration in multilayer treatments promises higher efficacy and safety. This approach marks a significant advancement in modern facial rejuvenation standards.

## 1 Introduction

Midface rejuvenation involves enhancing malar projection and restoring full cheek volume to preserve a youthful facial appearance, with beneficial effects on the nasolabial fold and the sub‐orbicular area. Given the multilayer structure of facial soft tissues, a multilayer approach to midface rejuvenation has already been proposed by Trévidic, who suggests treating both the deep and the superficial fat compartments of the midface using fillers with different rheological properties, including variation in elastic (G′) and viscous (G″) moduli, cohesivity, and swelling propensity. However, this approach does not address skin treatment, which remains a fundamental pillar in achieving a youthful appearance. Moreover, we believe that the integration of ultrasound guidance should not be overlooked in this type of treatment, as it offers significant advantages in ensuring the precise and safe placement of fillers across the different layers.

For these reasons, we propose that midface rejuvenation requires a structured approach grounded on three fundamental elements: a comprehensive understanding of anatomy, the use of facial ultrasound to guide precise injection techniques, and the selection of appropriate hyaluronic acid fillers, tailored to the three injection planes according to their rheological properties. The introduction of this three‐layer, eco‐assisted technique may represent a significant turning point in achieving safe and natural aesthetic results.

### 1.1 Elements of Anatomy

**FIGURE 1:**
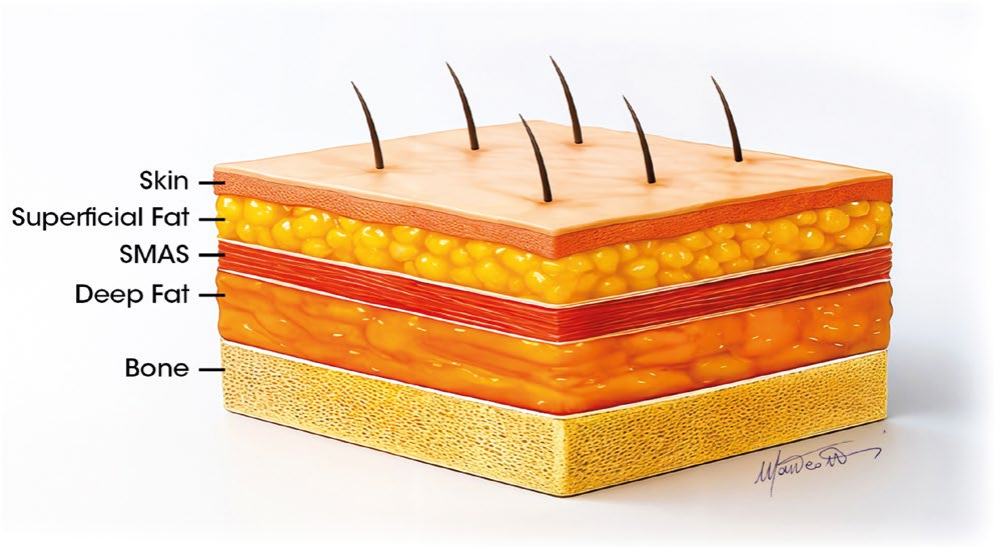
Anatomy of the multilayer structure in the midface.

To optimize procedural safety and achieve aesthetically consistent and natural results, clinicians must restore volume in the middle third of the face to master its three‐dimensional architecture. A key element of the multilayered treatment approach is understanding the five‐level structure of the face from bone to skin, which includes compartments of static deep fat and movable surface fat pads, separated by the superficial muscular aponeurotic system (SMAS) (Figure 1) [1, 2].

Another important aspect is the anatomy of the arterial vessels, which can vary significantly between individuals, making it essential to master this anatomy to avoid complications. This understanding guides clinical decisions regarding injection technique, depth of intervention, and precautions before and after every treatment.

Ultimately, it is crucial to recognize that the aging process affects each level, leading to interventions aimed at restoring a youthful appearance without compromising its natural integrity [3]. This section provides a comprehensive overview of the anatomy of the central face area, focusing on the structural and functional aspects for effective volumetric enhancement with fillers. Detailed anatomical studies reported in the scientific literature have revealed complex and variable configurations, often employing different terminologies for these structures.

#### 1.1.1 Superficial and Deep Fat Distribution

**FIGURE 2:**
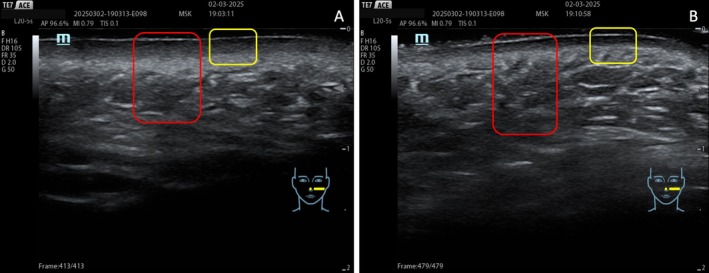
The thickness of the dermal and superficial compartments in a 26‐year‐old (A) and a 57‐year‐old patient (B). The dermal layer is highlighted in the yellow box, and the adipose layer is highlighted in the red box. The dermis is thinner, and the adipose layer is thicker in the older patient.

In the central region of the face, deep fat compartments firmly anchored to the bone provide essential structural support to the overlying soft tissue layers, including the Superficial Musculo Aponeurotic System (SMAS), the superficial fat, and the dermal–subcutaneous layers. The progressive atrophy of these compartments contributes to the collapse of the upper cheeks with age (Figure 2) [4, 5, 6]. The identification of the septa that separate the superficial fat compartments and those between the deep compartments is not possible without ultrasound visualization. However, identifying the SMAS is crucial to ensure the correct placement of products intended for the deep compartment, reducing the risk of superficial or intra‐SMAS injections and related complications.

As people age, volume loss in the deep panniculus adiposus reduces the medial projection of the midface, contributing to pseudoptosis due to excess skin.

**FIGURE 3:**
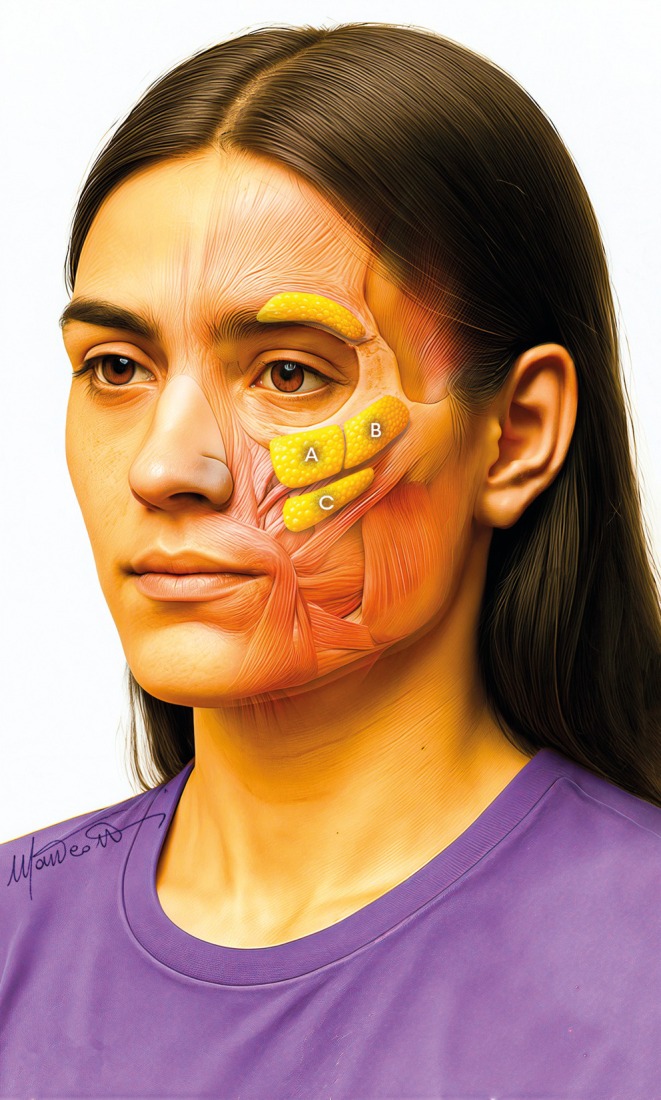
Deep compartments in the midface: (A) medial SOOF, (B) lateral SOOF, and (C) deep medial cheek fat.

**FIGURE 4:**
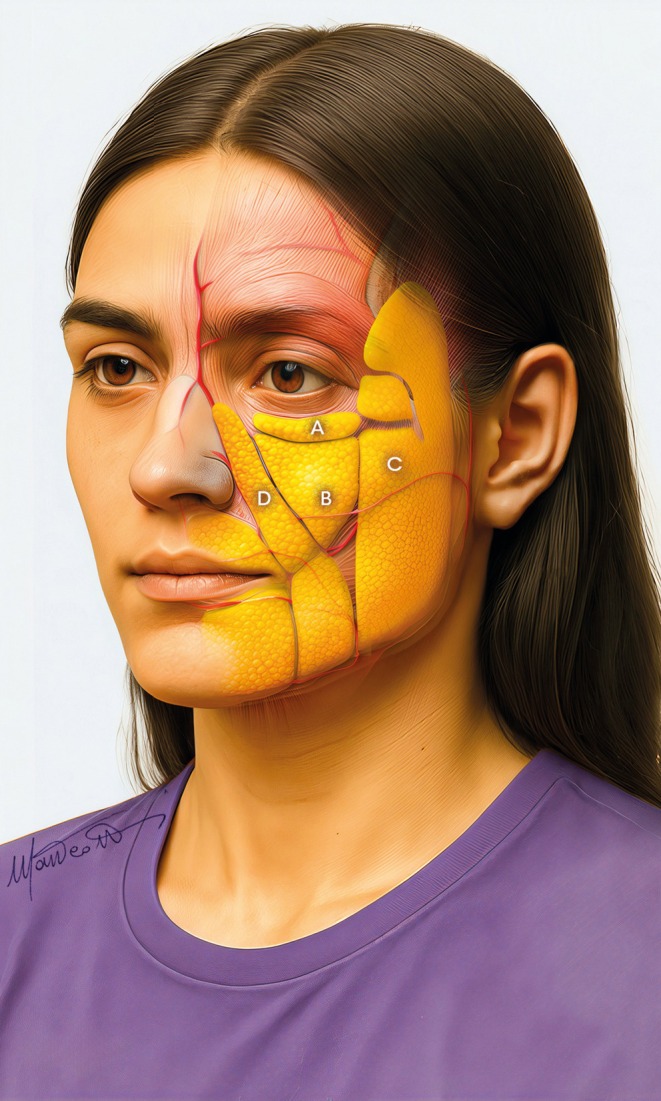
Superficial compartments in the midface: (A) Infraorbital fat compartment, (B) Medial cheek fat compartment, (C) Middle cheek fat compartment, and (D) Nasolabial fat compartment.

Therefore, it is necessary to focus on all fat compartments of the midface: the deep fat [(medial and lateral) suborbicularis oculi fat (medial‐ and lateral‐SOOF), and deep medial cheek fat (DMCF)] (Figure 3), and the superficial fat (infraorbital fat, medial cheek fat, middle cheek fat, and nasolabial fat) (Figure 4) [6, 7], whose treatment is planned according to the multilayer technique [8, 9].

Recent anatomical investigations reveal that the SOOF and DMCF are systematically divided into separate, easily identifiable compartments [7]. In addition, these structures are permanently adhered to the bone; therefore, their treatment with filling techniques must be targeted and precise.

#### 1.1.2 Vascular Anatomy

In clinical practice, anatomical references are used to outline areas known as “safety zones,” which are defined by tracing a straight line from the medial canthus to the mandibular angle (designated as the medial cheek safety line) and a second line that descends vertically from the center of the zygomatic arch (referred to as the lateral safety line) [8].

**FIGURE 5:**
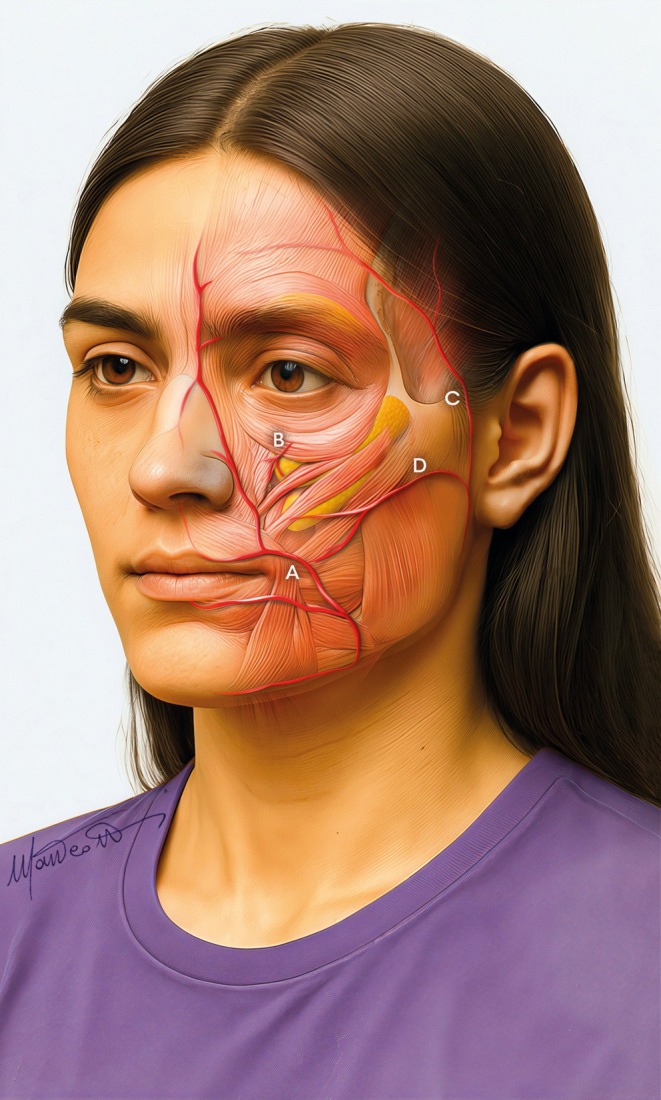
Arterial network in the midface: (A) Facial artery, (B) Infraorbital artery, (C) Temporal artery, and (D) Transverse facial artery.

The facial artery (FA), a major vessel that supplies blood to the face, originates from the external carotid artery and extends to the facial region through the submandibular area, passing anterior to the masseter muscle. As demonstrated by previous studies, this artery displays considerable variability in its path among individuals (Figure 5) [10, 11, 12, 13].

A recent study based on cadaveric anatomical analyses showed that FA mainly follows the nasolabial sulcus, entering the angular artery in 36.6% of cases, the lateral nasal artery in 48.6%, and the superior labial artery in 8.5%. Interestingly, a hypoplastic variant of FA ends in the inferior labial artery in 6.8% of individuals. Additionally, about 30% of the population exhibits a bifurcation of the AF into a nasolabial trunk and an infraorbital trunk, which extends vertically toward the nasojugal fold [10, 11, 12, 13].

At the same time, a recent ultrasound study [14] shows significantly different percentages concerning the course of the FA tract, identified as the Angular artery. This highlights the variability in vessel distribution related to tissue thickness and lateral‐medial vessel dislocation.

Given this anatomy, it is crucial to take proper precautions during invasive procedures and to monitor any symptoms that may arise.

The infraorbital artery, a vital vessel in the middle third, also needs to be evaluated. It arises from the infraorbital foramen and branches into two or three main branches: the palpebral, nasal, and labial branches. The latter two are located within the DMCF and supply blood, respectively, to the lateral nasal and upper labial areas, mainly below the safety line of the medial cheek [15]. It is important to note that the infraorbital foramen points downwards toward the mandible; therefore, it is best to approach this area from below at an acute angle to reduce the risk of arterial injury [16].

In treatments targeting the middle third of the face, evaluating the Transverse Artery of the Face (TAF) is crucial, as it represents a key vascular structure that supplies the cheek region [17]. Anatomical studies have demonstrated that the majority of individuals have a single TAF, which arises from the superficial temporal artery and splits into an upper and a deeper branch. The two branches supply the parotid gland and its duct as well as the masseter muscle [18].

During ultrasound‐guided procedures, careful attention to the superior emergent branch is essential. This branch runs into the subcutaneous tissue and poses a risk of cannulation during filler injections into the superficial fat of the lateral cheek [19].

Moreover, the Zygomatic–Facial artery (ZFA) must also be assessed. This vessel originates within the SOOF, arising from the lacrimal artery and emerging through the zygomatic–facial foramen. Although this foramen has rarely been described, studies have localized it laterally to the lateral canthus [20, 21, 22, 23]. It has also been observed that both the ZFA and the foramen may be absent in some patients, making careful ultrasound evaluation in this region fundamental.

Lastly, the facial vein, easily recognizable by its origin and course, functions as the main venous drainage vessel for the face. The facial vein, located along the lower edge of the mandible, courses lateral to the FA and proceeds directly toward the medial canthus, passing beneath the zygomaticus major and minor muscles. Since the FA lies beneath the medial cheek slit, it is located below the medial cheek safety line [18].

### 1.2 High‐Frequency Facial Ultrasound

Given these anatomical factors, the integration of ultrasound into clinical practice becomes a critical tool for both assessment and accurate filler placement and distribution.

Ultrasound assessment is pivotal throughout all stages of filler treatment, including pre‐procedural planning, intra‐procedural guidance, and post‐procedural evaluation.

#### 1.2.1 Before the Procedure

In facial rejuvenation procedures, a pre‐procedure ultrasound examination can facilitate a more accurate distinction between a youthful and a mature face, evaluating the thickness of the dermis and the fat layers. With aging, the dermis becomes progressively thinner, while the fat layer becomes progressively thicker (Figure 2) [24, 25].

Ultrasound examination can also allow the visualization of anatomical morphology and tissue dynamics, facilitating the accurate identification of both superficial and deep fat compartments, the positioning of muscles, and the pathways of the main blood vessels and nerves. It enables the creation of a precise anatomical map, which would remain theoretical without ultrasound guidance.

Moreover, ultrasound mapping facilitates the planning of injection pathways in targeted areas, thereby avoiding high‐risk zones and reducing the likelihood of intravascular injection or damage to surrounding tissues [26, 27]. Ultrasound examination enhances the identification of the SMAS, which appears as a hyperechoic structure measuring several millimeters in thickness, separating the superficial from the deep compartments [28]. Recognizing the SMAS allows for precise identification of the injection area, whether superficial or deep, preventing filler injections into the SMAS, thereby reducing the risk of complications such as inflammatory nodule formation or unwanted product migration [29, 30].

Considering all the above, pretreatment ultrasound evaluation helps delimit safe areas and select the optimal access route to the target areas.

#### 1.2.2 During the Procedure

During treatment, ultrasound assists the doctor in identifying (a) the different layers for filler deposition, (b) the areas to avoid for optimal results, and (c) the critical high‐risk zones, especially for preventing complications. Ultrasound‐guided filler injection is crucial for accurately locating the injection sites, monitoring filler distribution during the procedure, and assessing post‐procedural integration to ensure the correct product is administered in the intended location.

**FIGURE 6:**
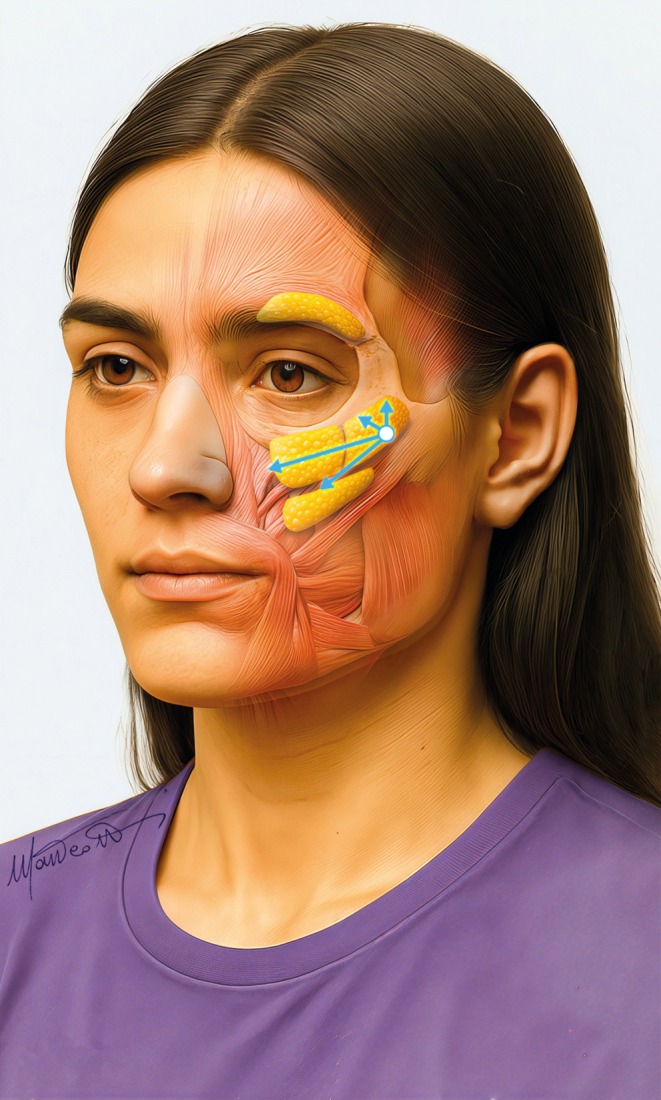
Superior–lateral entry point to access the superior deep compartments (medial and lateral SOOF).

**FIGURE 7:**
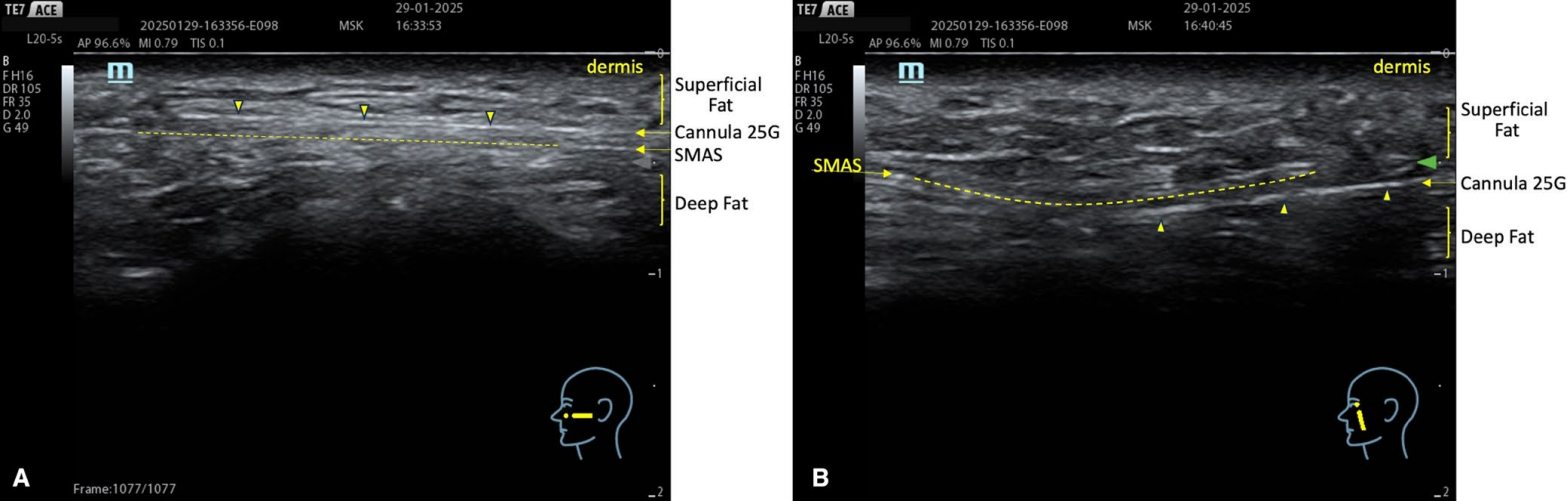
(A) Accessing the deep compartments with a cannula from a superior entry point is impossible. The cannula will stay in the superficial zygomatic compartment. (B) Accessing the deep compartments beneath the SMAS is possible by choosing an inferior entry point (B).

**FIGURE 8:**
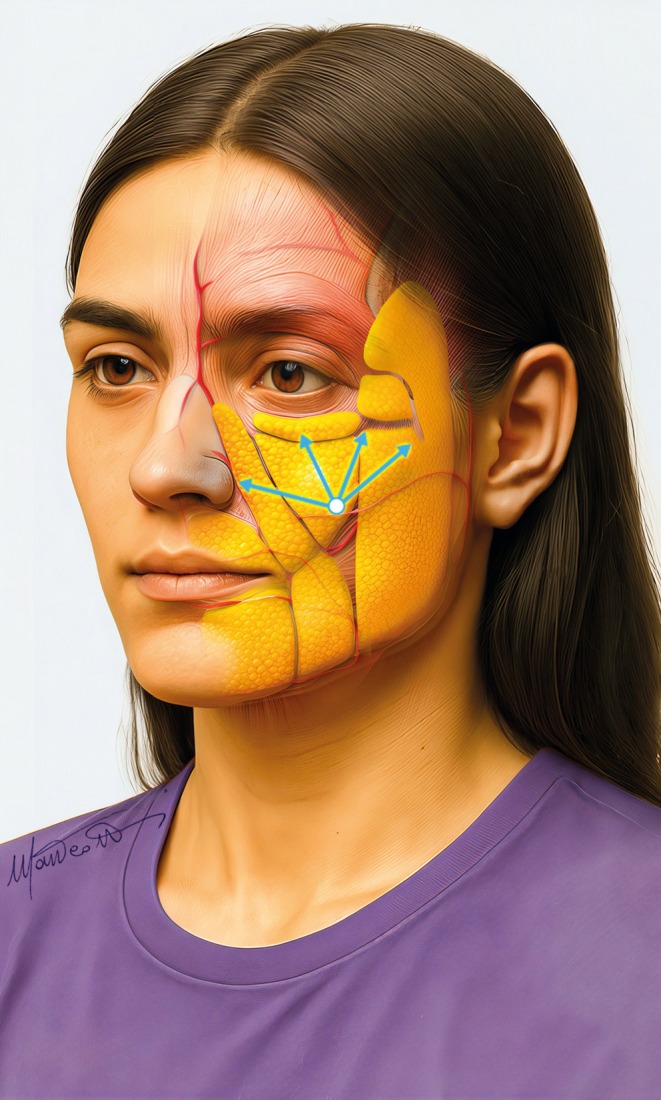
Inferior entry point to easily access both superficial and deep compartments of the midface.

**FIGURE 9:**
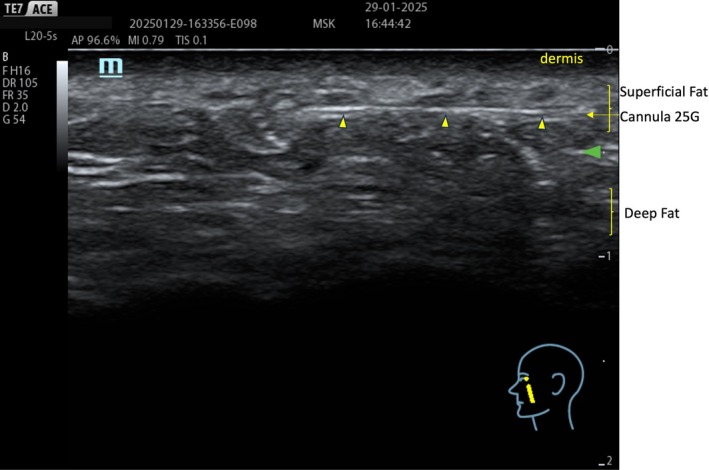
An inferior entry point makes accessing superficial planes easier.

**FIGURE 10:**
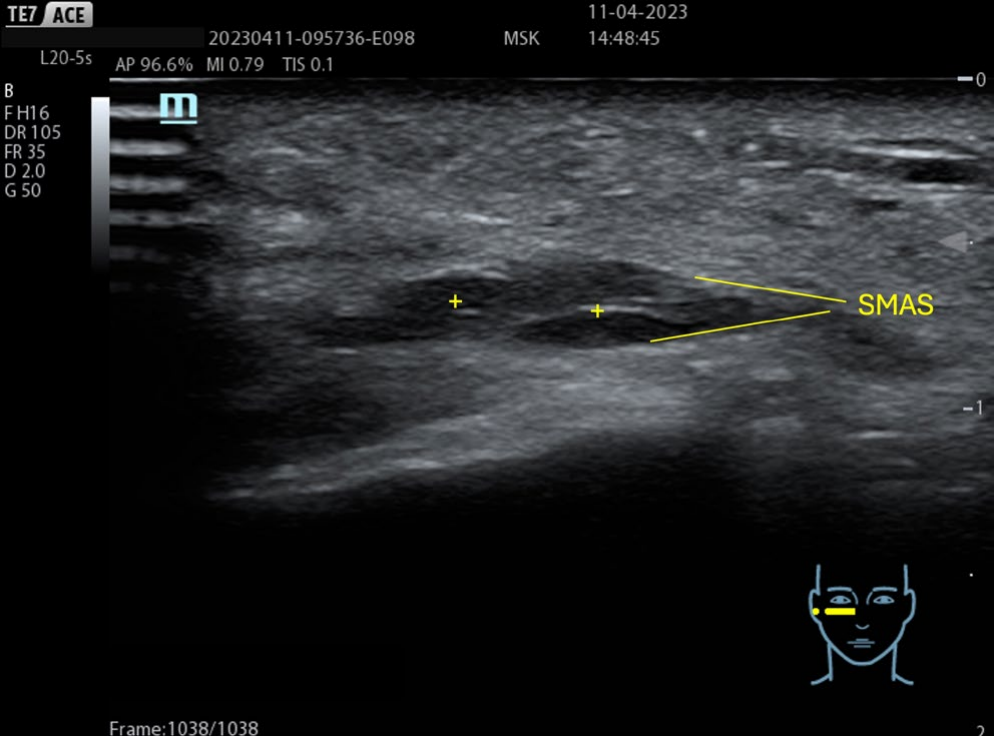
HA deposition (+) within the SMAS is possible when the treatment is performed without ultrasound guidance.

**FIGURE 11:**
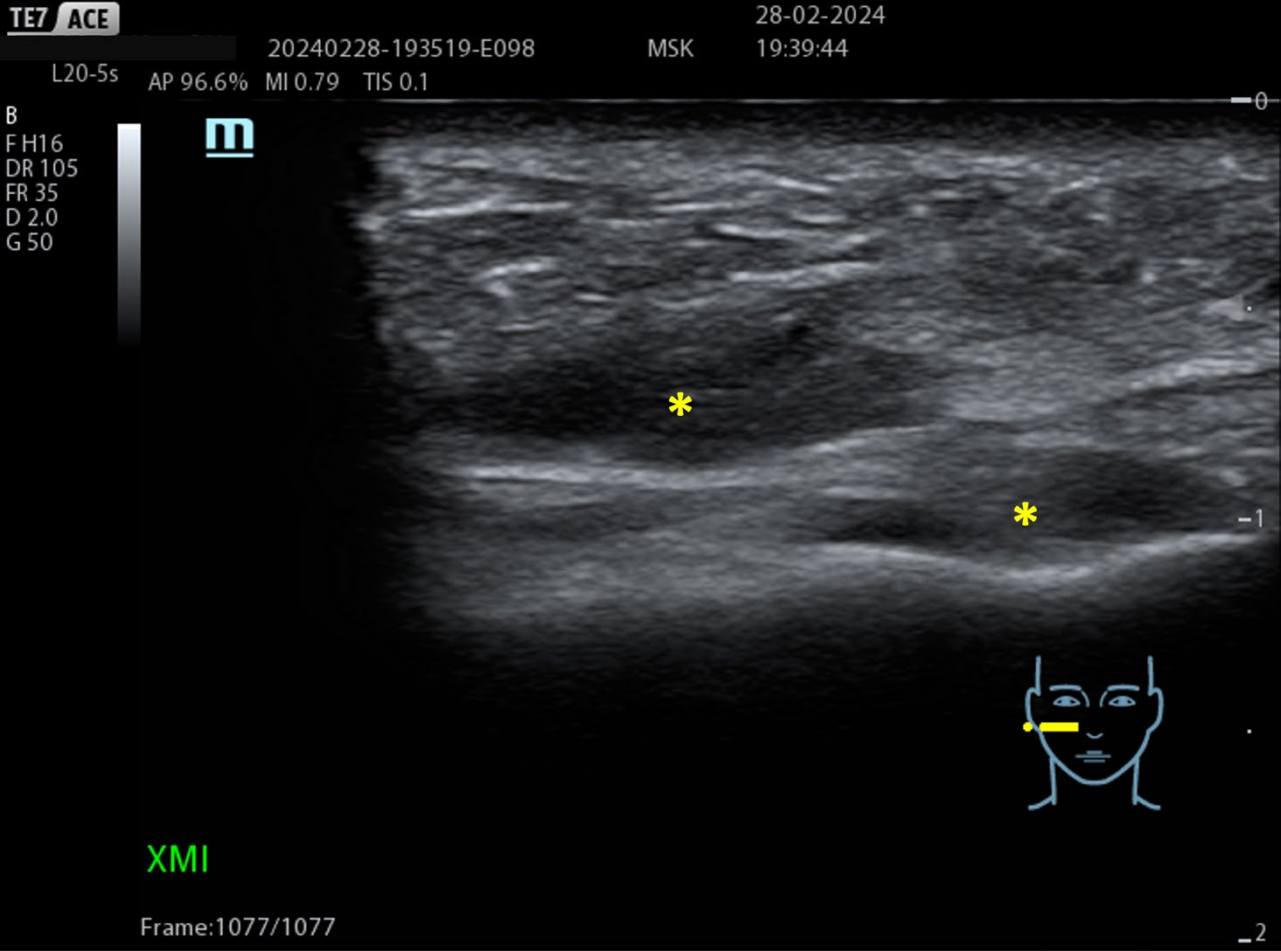
Hydrophilic deposits (+) of the same viscoelasticity in both deep and superficial compartments.

For example, only through ultrasound guidance is it possible to realize that, when treating the deep fat compartment of the midface, filler injections into the deep space are not feasible via the commonly used superolateral access (Figure 6) due to the compartment's depth and the high flexibility of the cannula (Figure 7). Thus, when treating this compartment with a cannula, only a low approach (Figure 8) ensures the injection in the target layer. The same result could be achieved with an approach from the zygomatic arch, only with a needle. Simultaneously, a lower approach facilitates injections into the superficial compartments of the middle third (Figure 9). Finally, a correct identification of SMAS and fat layers during injection prevents fillers from being inadvertently deposited in the SMAS (Figure 10) or, simultaneously, in the superficial and deep compartments (Figure 11).

#### 1.2.3 After the Procedure

After treatment, ultrasound support enables real‐time verification of the accurate distribution of the filler and, over time, completes the evaluation of outcomes. Indeed, ultrasound analysis provides visual evidence of optimal product integration and permanence, highlighting the significance of facial ultrasound in monitoring procedure execution as well as in the long‐term assessment of outcomes.

### 1.3 Fillers

Careful selection of fillers represents a crucial aspect of the therapeutic approach to midface rejuvenation. The choice of the correct hyaluronic acid (HA) filler must rely on its specific rheological properties, which are designed to integrate within various tissue compartments, including both superficial and deep layers. This helps achieve long‐lasting aesthetic results while maintaining a natural look during dynamic expressions, such as smiling [9, 30]. Although the effectiveness of HA fillers for midface rejuvenation is well known, few have outlined a systematic multilayer approach that matches fillers with specific rheological properties to targeted tissue compartments [30].

Since deep fat compartments are anatomically distinct from septa and ligaments, while superficial compartments exhibit less segmentation, selecting the most suitable filler for specific compartments is a crucial key to preventing undesirable effects, such as filler migration and visible nodules.

In modern filler assessment, viscoelastic properties are measured using rheological parameters such as elastic modulus (G′) and viscous modulus (G″), which determine the filler's hardness and resistance to deformation. These parameters are important, but not the only factors to consider. Other mechanical parameters that have been recently defined [30], such as strength and stretch scores, must also be considered to address the limitations of measuring G′ under quasi‐static conditions. More specifically, the strength of a gel indicates its ability to maintain its characteristics under a wide range of mechanical stresses, while the stretch describes its tendency to deform and adapt to mechanical constraints, such as those in response to the endless facial movements [30]. The application of these fillers, called “resilient”, is essential for the harmonious remodeling of both deep and superficial compartments, particularly in patients with variable skin thickness, whether thin or thick. These fillers, characterized by a softer and more moldable consistency, exhibit slightly lower strength but greater stretchability. These properties allow better adaptation to facial movements and help minimize the risk of visible irregularities [9]. Fillers considered “firm”, namely those characterized by high G′ and significant strength, are ideal for injections in the deep static adipose compartment: the filler should be placed at bone contact, where significant structural support and pronounced zygomatic projection are required. In individuals with thinner skin, softer and more moldable fillers with slightly lower strength but greater stretch are preferable to avoid the appearance of visible irregularities [9].

Unlike cross‐linked fillers, which incorporate cross‐linking agents to create a more stable, gel‐like matrix for volumizing effects, non‐cross‐linked hyaluronic acids are specifically designed for application within the dermal layer. They are classified as skin boosters and are more fluid to spread in the target layer, providing hydration, thus improving skin quality and luminosity. These products are often combined with essential nutrients, such as amino acids, vitamins, and minerals, which help stimulate collagen and elastin synthesis to boost dermal density.

## 2 Methods

**FIGURE 12:**
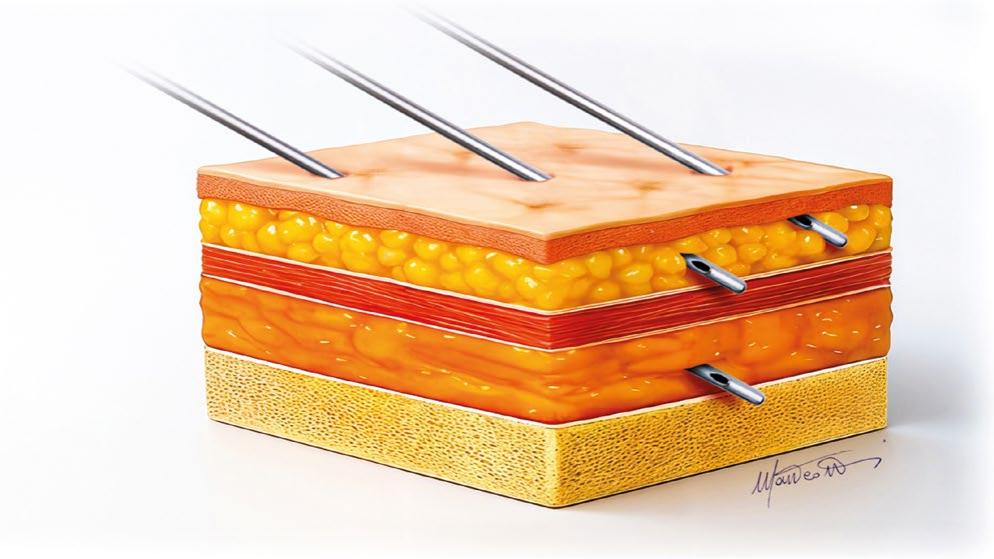
The cannulas showing the correct position in the three compartments to be treated.

The new three‐layer ultrasound‐guided approach we propose for midface rejuvenation, as its name suggests, targets the reported three facial compartments (Figure 12) to improve both procedural accuracy and treatment outcomes.

### 2.1 Deep Fat

Treatment begins with supraperiosteal injections targeting the deep fat folds, primarily within the fat compartments beneath the inferior orbital rim, such as medial and lateral SOOF, SOOF, and DMCF. In this area, “firm” fillers with high projection capacity are preferred, such as Teosyal PureSense Ultra Deep (Teoxane, Rue de Lyon 105, 1203 Geneva, Switzerland). Firm fillers in deep fat compartments aim to provide structural support for a solid foundation and enhance malar projection. A low entry point, using a cannula, or a superolateral entry point with a needle, is used for targeting this plane (Figures 6 and 8).

### 2.2 Superficial Fat

The second phase involves injections into the superficial fat compartments using more elastic, moldable fillers, such as Teosyal RHA4 or Teosyal RHA3 (Teoxane, Rue de Lyon 105, 1203 Geneva, Switzerland). These fillers, directly injected into superficial compartments, help maintain a natural look and allow for dynamic facial expressions. Their specific integration in the tissue prevents nodule formation and product migration.

### 2.3 Subdermal Infiltration

The treatment concludes with subdermal infiltrations of non‐cross‐linked products such as Teosyal PureSense Redensity 1 (Teoxane, Rue de Lyon 105, 1203 Geneva, Switzerland). This type of filler, when applied in the subdermal layer, effectively restructures the deep dermis, improving skin density and appearance [31]. These layers are approached by the same low approach (Figure 8), but the cannula must be directed in the appropriate plane at a more acute angle.

Fifteen patients (13 women, two men, age 50 ± 8 years old) (range 42–65) were treated with the reported technique in one shot. They were controlled after 1 week and at 3 months distance to assess the midterm outcome. Inclusion criteria: patients in the age range 40–70, both sexes, without previous filler treatment in the treated area, in a healthy status, without systemic disease. Exclusion criteria: patients younger than 40 years and older than 70 years, with systemic diseases or taking antiplatelet or anticoagulant medications, pregnant, or breastfeeding. The treated patients were informed about the given treatment and signed an informed consent to be treated.

## 3 Results

**FIGURE 13:**
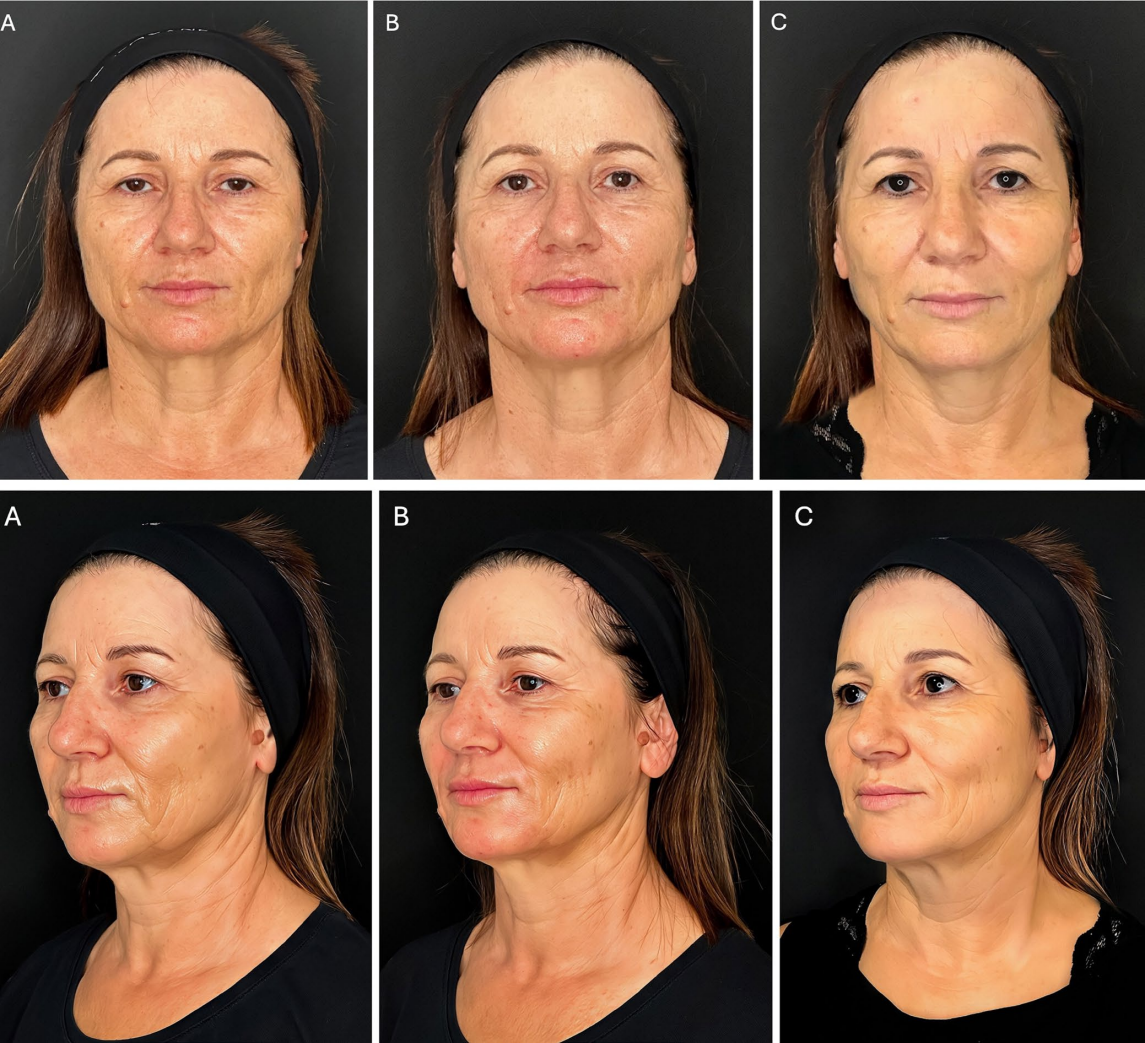
Front and three‐quarter views of a 56‐year‐old female. (A) Pretreatment: Patient with an aged face, showing tissue ptosis and skin laxity. The cheeks are hollowed out, and age furrows, visible in all projections, mark the skin, which is dull, devitalized, dehydrated, and uneven. (B) Post‐3‐layer midface treatment: The cheeks are immediately filled, lifted, and elevated, leading to noticeable improvements in all projections. Both frontal and three‐quarter views show the effect of indirect lifting on the face's oval, caused by the traction from the volumetric increase of the malar area. The skin also appears visibly better—more relaxed, brighter, and hydrated, with a noticeable improvement in surface smoothness and furrow depth. (C) Eight months after treatment: The filler is still completely integrated into the tissues, and the result remains evident: The cheeks are filled with a homogeneous volume, the cheekbones are well‐projected, and the lifting effect is still visible. The face remains overall rejuvenated, with harmonious proportions, and the skin appears glowing, relaxed, and more compact.

**FIGURE 14:**
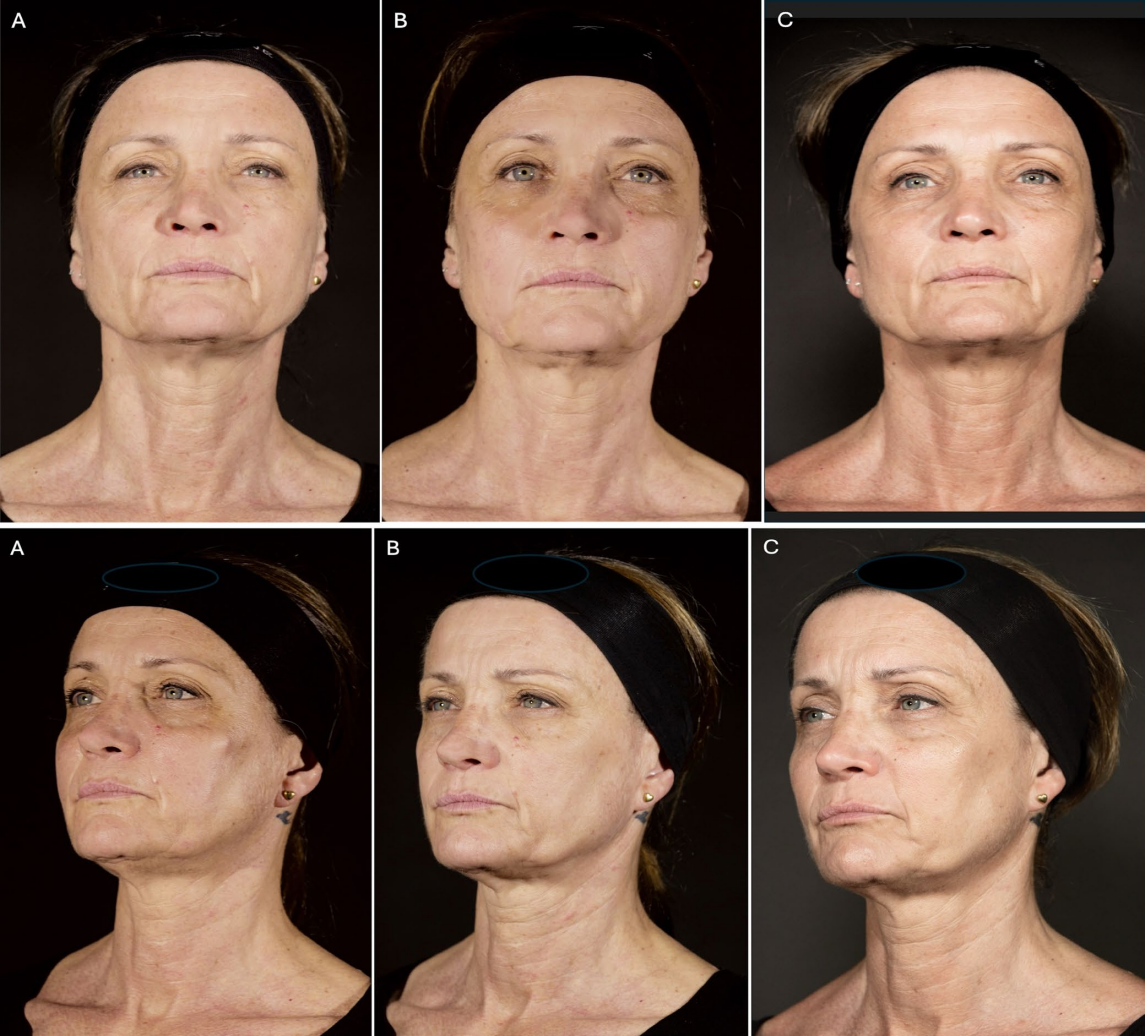
Front and three‐quarter views of a 57‐year‐old female. (A) Pretreatment: Patient with a tired face, with skin showing aging signs, in which tissue ptosis and skin laxity are evident (especially at three‐quarters and laterally). The cheeks are emptied, the tissues have slipped down, and the skin is moderately marked, dehydrated, dull, and devitalized, which is evident in all projections. In addition, especially in lateral viewing, there is a flat zygomatic bone and a lack of zygomatic prominence. (B) Post‐3‐layer midface treatment: The cheeks are immediately filled and lifted with a fresh and rested appearance. In the three‐quarter view, a moderate improvement in the zygomatic prominence can be appreciated. The skin is also improved, bright, more hydrated, and relaxed. (C) Seven months after treatment: The filler is integrated into the face tissues, and the aesthetic result is even more evident, especially concerning the lifting and projection effect. The skin is also visibly improved, more relaxed, glowing, compact, and hydrated. The face is overall rejuvenated, fresh, and rested.

The treatment resulted in benefits for all the treated patients. In particular, the cheeks resulted in immediate filled and lifting with a more prominent zygomatic arch. At the first control, the skin appeared brighter, hydrated, smoother, and relaxed. No side effects were reported (Figures 13 and 14).

**FIGURE 15:**
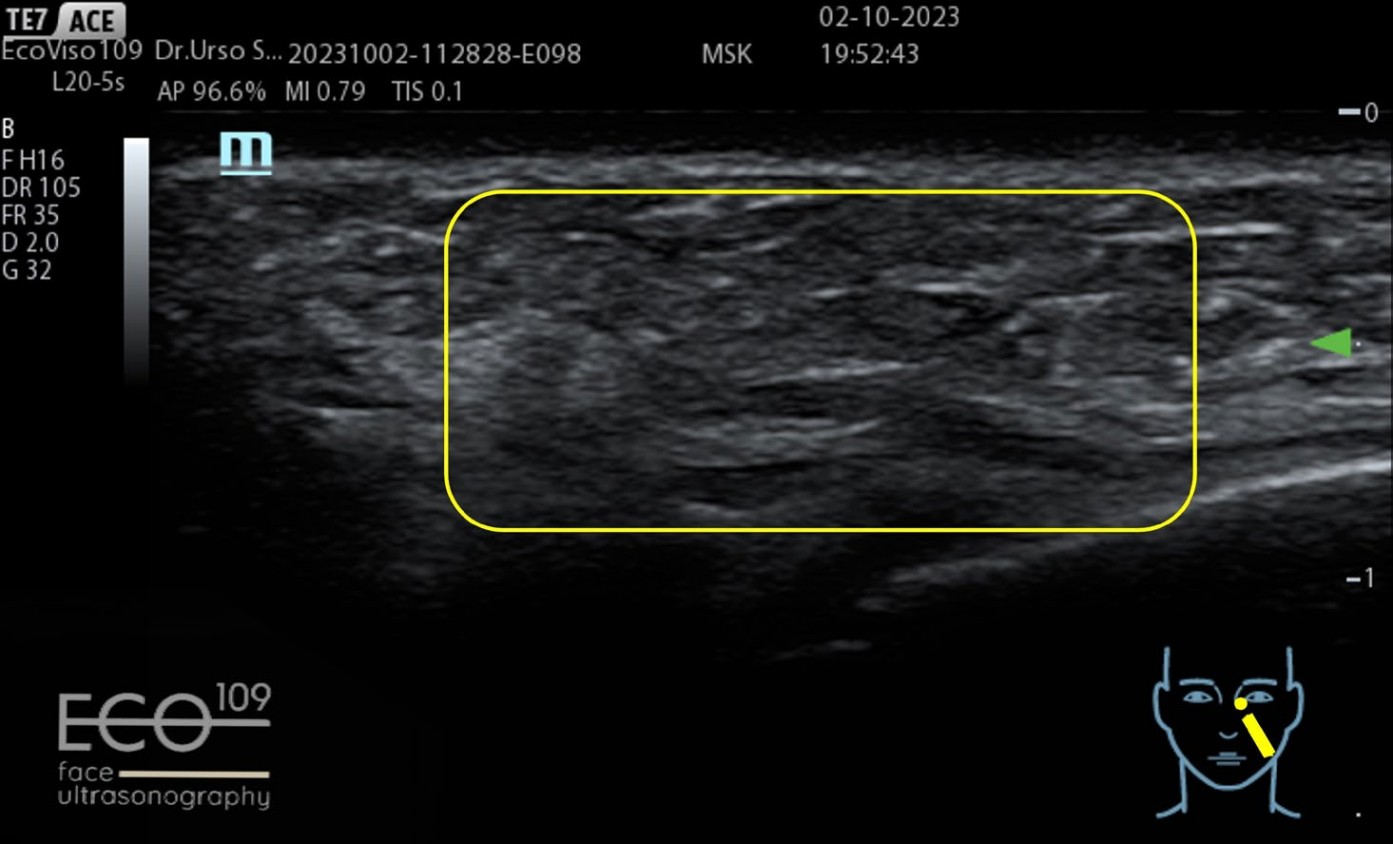
Before: Normal dermal and subcutaneous ultrasound stratification is observed.

**FIGURE 16:**
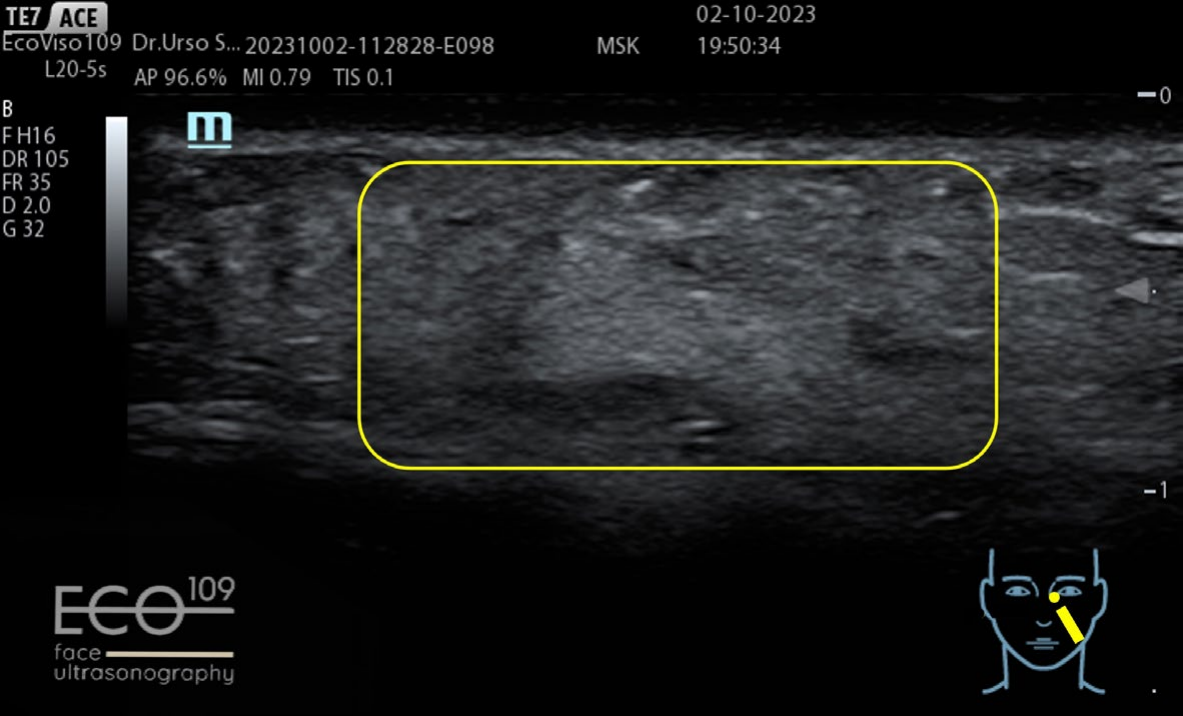
After: A homogeneous and uniform echogenic pattern is visible within the subcutaneous layer, indicating a recent injection of a hydrophilic, fully absorbable filler with high tissue integration, with no evidence of fragmentation or encapsulation.

The ultrasound examination revealed the filler deposition in the correct anatomical plane, its perfect filler integration in the patient tissue without any fragmentation (Figures 15 and 16).

## 4 Discussion

The multilayer technique we propose as a proof‐of‐concept involves injecting different fillers into specific facial compartments. Selecting the proper filler is essential and must be customized to the patient's unique features, including bone structure, skin thickness, and the degree of volumetric loss.

This method can rebalance the volume in the mid‐third of the face, restoring a natural appearance (Figures 13 and 14) while simultaneously enhancing skin quality. Questions may arise regarding the optimal method for treating the most superficial layer, as it is typically addressed with intradermal injections using very thin needles, following the nappage technique.

In our method, the non‐cross‐linked filler is injected into the subdermal layer via a cannula. We hypothesize that when administered in this manner, this product may prolong the effects of deeper fillers beyond its hydrating effect; however, further studies are required to confirm this hypothesis.

Another practical benefit of this technique, even if it lacks scientific significance, is that the injection is made at the same entry point as the previous treatment. This helps avoid multiple intradermal injections, which can cause discomfort to the patient and an unpleasant appearance for several hours.

Compared to single‐plane injections, the three‐layer technique offers enhanced effectiveness, addressing all aspects of facial aging while maintaining a natural look. Therefore, this approach is particularly well‐suited for patients with a hollow face or mature skin, as it provides the necessary projection, preserves natural mobility and expressiveness, and improves skin quality. In contrast, single‐plane filler injections, which mainly target deep fat compartments, are generally indicated for younger patients with minimal volumetric loss.

The introduction of ultrasound as a supportive tool in treatments with HA fillers marks a significant advancement: in the pretreatment phase, enabling real‐time visualization of anatomical structures; during treatment, enhancing accuracy by injecting the proper filler into the target space while reducing associated risks and complications; in the posttreatment phase, allowing real‐time monitoring of the interaction between the filler and surrounding tissues.

Integrating ultrasound into daily practice requires adequate operator training; however, the benefits in treatment safety and efficacy are undeniable.

Though ultrasound assessment is not yet integrated into clinical practice, particularly within the multilayer technique, current evidence demonstrates its potential to elevate the standards of modern aesthetic medicine.

A limitation of the present work lies in the absence of well‐structured data derived from a large case series. As the ultrasound‐guided three‐layer technique represents a novel approach, such comprehensive datasets are not yet available. However, the really encouraging results coming from this preliminary study convinced us to treat a substantial number of patients consistently demonstrating favorable outcomes, including concurrent improvements in skin quality and midface volumetric restoration. With the wider clinical adoption of this technique, it is anticipated that a larger patient cohort will be treated, thereby enabling more rigorous validation of our preliminary observations. The data coming from this case series are under close examination and will be the object of a new publication.

## 5 Conclusions

Treating the three layers of the midface simultaneously (including the deep and superficial fat pads alongside the dermal layers), combined with the careful selection of fillers tailored to their specific rheological properties for each tissue compartment, represents a significant advancement in facial rejuvenation. Performed under ultrasound guidance, this approach enhances procedural precision and efficacy while maximizing safety, thereby setting new standards in aesthetic medicine.

## Funding

The authors have nothing to report.

## Ethics Statement

This is an observational study. For this kind of study, Italian Authorities do not request an Ethical Committee approval. The study was performed according to the Helsinki Declaration principles.

## Consent

The patients who received the new three‐layer treatment were fully informed about the treatment and gave their informed consent.

## Conflicts of Interest

Paola Molinari, Simone Ugo Urso, and Giovanni Mosti wrote the manuscript and checked the references; Chiara Faso controlled the manuscript, especially regarding the English form, and suggested some changes to make its reading more fluent; Ilenia Iacovone helped us with the reference research and verified that the details regarding the used products are correct. All authors declare no conflicts of interest regarding their author contributions.

## Data Availability

The data that support the findings of this study are available on request from the corresponding author. The data are not publicly available due to privacy or ethical restrictions.
